# Multiple Insulin Degrading Enzyme Variants Alter *In Vitro* Reporter Gene Expression

**DOI:** 10.1371/journal.pone.0021429

**Published:** 2011-06-22

**Authors:** Olivia Belbin, Michael Crump, Gina D. Bisceglio, Minerva M. Carrasquillo, Kevin Morgan, Steven G. Younkin

**Affiliations:** 1 Department of Neuroscience, Mayo Clinic College of Medicine, Jacksonville, Florida, United States of America; 2 School of Molecular Medical Sciences, Institute of Genetics, Queen's Medical Centre, University of Nottingham, Nottingham, United Kingdom; Naval Research Laboratory, United States of America

## Abstract

The insulin degrading enzyme (*IDE*) variant, v311 (rs6583817), is associated with increased post-mortem cerebellar *IDE* mRNA, decreased plasma β-amyloid (Aβ), decreased risk for Alzheimer's disease (AD) and increased reporter gene expression, suggesting that it is a functional variant driving increased *IDE* expression. To identify other functional *IDE* variants, we have tested v685, rs11187061 (associated with decreased cerebellar *IDE* mRNA) and variants on H6, the haplotype tagged by v311 (v10; rs4646958, v315; rs7895832, v687; rs17107734 and v154; rs4646957), for altered *in vitro* reporter gene expression. The reporter gene expression levels associated with the second most common haplotype (H2) successfully replicated the post-mortem findings in hepatocytoma (0.89 fold-change, p = 0.04) but not neuroblastoma cells. Successful *in vitro* replication was achieved for H6 in neuroblastoma cells when the sequence was cloned 5′ to the promoter (1.18 fold-change, p = 0.006) and 3′ to the reporter gene (1.29 fold change, p = 0.003), an effect contributed to by four variants (v10, v315, v154 and v311). Since IDE mediates Aβ degradation, variants that regulate *IDE* expression could represent good therapeutic targets for AD.

## Introduction

Aggregated β-amyloid (Aβ) is a pathological hallmark of the Alzheimer's Disease (AD) brain. Mechanisms that alter the production and clearance of toxic Aβ in the brain therefore represent good therapeutic targets for prevention and/or treatment of AD. Insulin degrading enzyme (IDE), one of several Aβ-degrading proteases [1]-[4] identified to-date, has been shown to mediate Aβ degradation *in vitro* [5]–[7] via an IDE recognition motif corresponding to residues 17–24; sites crucial for the formation of Aβ fibrils. Furthermore, Farris *et al.* reported that IDE knock-out mice presented with a >50% decrease in Aβ degradation in both brain membrane fractions and primary neuronal cultures and increased cerebral accumulation of endogenous Aβ [8]. It follows that disturbed IDE expression and/or activity could contribute to amyloid fibril formation [9].

The gene encoding IDE (*IDE*), located on chromosome 10q23.33, represents a strong AD candidate gene because of its location near a “suggestive” linkage peak (10q24) found in two genome-wide linkage studies of late-onset AD (LOAD) families [10], [11] and a “significant” linkage peak (10q) in a third [12]. Several studies have reported significant association of *IDE* haplotypes with LOAD [13], [14], [15], as well as with plasma Aβ42 levels in extended LOAD families [14], with plaque density [13] and cognitive function [14].

In our recent publication we identified three *IDE* haplotypes (H2, H6 and H9) associated with altered *IDE* transcript levels in post-mortem cerebellum [15]. A summary of the composition of these haplotypes and our previous findings are shown in Table 1. Since rs6583817 (v311), a variant present on both H6 and H9, was individually associated with an increase in the level of *IDE* transcript, increased *in vitro* reporter gene expression and decreased risk of LOAD, we proposed a simple model in which v311 is the functional variant that influences *IDE* expression and LOAD risk. This was supported by the finding that rs5786996 (v3), a variant tagging H9, although associated with increased *IDE* transcript and decreased risk for LOAD, showed a conflicting decreased *in vitro* reporter gene expression and was thus unlikely to be the functional variant driving increased transcript levels. However, the observation that the ∼1.4-fold increase *in vitro* previously we reported for v311 did not compare to the 2.1-fold increase we observed in post-mortem cerebellum samples could indicate that rather than a single variant model for functionality, a combination of relatively small effects on gene expression resulting from multiple variants may produce larger and significant changes in gene expression.

**Table 1:** Summary of 17 *IDE* variants and haplotype composition.

Single variantinformation								Haplotypecomposition			Variant mRNA association	
vID	rs	Maj	Min	Position	Location	Cons.	MAF	H2	H6	H9	Δ	p
2	-	AA	--	94202383	3′	81%	2%	0	0	0		
3	rs5786996	-	C	94202516	3′	74%	4%	0	0	1	2.12	0.0005
6	rs5786997	--	AT	94203071	3′	88%	10%	0	0	0		
10	rs4646958	A	T	94204339	Int24	75%	9%	0	1	0	2.2	4.2×10^−6^
154	rs4646957	G	A	94219892	Int18	71%	36%	0	1	1	1.22	0.02
309	-	G	T	94236972	Ex13	92%	2%	0	0	0		
310	-	T	C	94237153	Int12	72%	2%	0	0	0		
311	rs6583817	G	A	94237227	Int12	74%	13%	0	1	1	2.12	1.5×10^−8^
176	rs17875327	T	C	94264789	Int4	85%	10%	0	0	0		
315	rs7895832	T	A	94266506	Int4	83%	9%	0	1	0	2.19	6.3×10^−6^
46	rs4646955	T	C	94284271	Int3	76%	25%	0	0	0		
684	rs17107721	G	A	94288480	Int1	71%	5%	0	0	0		
180	rs11187060	G	A	94294112	Int1	70%	33%	0	1	1	1.22	0.02
683	rs17445328	A	G	94295169	Int1	72%	25%	0	0	0		
685	rs11187061	C	T	94295389	Int1	70%	19%	1	0	0	0.78	0.009
687	rs17107734	C	T	94295397	Int1	70%	9%	0	1	0	2.07	1.8×10^−5^
776	rs11187074	C	G	94316926	Int1	73%	19%	1	0	0	0.80	0.02
				Haplotype Frequency				18%	8%	3%		
				Haplotype mRNA association			Δ	0.79	2.24	2.34		
							p	0.03	1×10^−5^	0.006		

The variant ID (vID), rs number (where available), major (Maj) and minor (Min) allele, Chromosomal position, location within the gene, % conservation of surrounding sequence with mouse and rat genomes and minor allele frequency (MAF) are detailed for each variant. The allele composition of the three haplotypes studied here are defined as 0 = major allele, 1 = minor allele. The fold-difference (Δ) in cerebellar *IDE* mRNA levels are given for each individual variant and haplotype that demonstrated significance at the p<0.05 level only.

In order to thoroughly investigate other potentially functional *IDE* variants we have tested five variants for altered *in vitro* reporter gene expression, based on their presence on haplotypes (H2, H6 and H9) and their association with altered *IDE* mRNA transcript levels in post-mortem cerebellum. Firstly, we evaluated a variant, rs11187061 (v685), present on H2, a haplotype associated with significantly decreased cerebellar *IDE* mRNA levels. Secondly, three variants (rs4646958; v10, rs7895832; v315 and rs17107734; v687) that are in near complete linkage disequilibrium (LD; all pair-wise D'<98, r^2^>90) and arise on one (H6) of the two haplotypes tagged by v311. Due to this strong LD, all three variants and v311 have near identical significant association (2.07–2.20 fold increase) with cerebellar *IDE* mRNA levels and all lie in conserved intronic regions of *IDE.* Finally, we also tested rs4646957 (v154), the most frequent of these *IDE* variants, which is present on five *IDE* haplotypes, two of which (H6 and H9) showed significantly increased cerebellar *IDE* mRNA levels.

## Methods

### Cloning

Approval was obtained from the ethics committee and institutional review board for the ascertainment and collection of samples (Mayo Clinic College of Medicine, Jacksonville). Written informed consent was obtained for all individuals that participated in this study. DNA sequence fragments surrounding each variant were each cloned into a pGL3P vector (Promega) containing the *Firefly Luciferase* gene and an SV40 promoter using the Gateway cloning system (Invitrogen). AttB-flanked primers (Table 2) specific to the region surrounding the fragments were used to amplify genomic DNA extracted from individuals known to be homozygous for the major or minor allele of each variant. PCR reactions were performed in a reaction mix containing 1xPCR buffer containing 1.5mM MgCl2 (QIAGEN), 1 mM dNTPs (Promega), 0.2 µM each primer, 2.5U HotStar Taq DNA polymerase and 20 ng genomic DNA to a final volume of 25 µl. Amplification conditions were as follows; 95°C for 5 minutes, followed by 35 cycles of 95°C for 30 seconds, annealing temperature (Table 2) for 1 minute, 72°C for 1 minute and finally an extension step of 72°C for 10 minutes. The resultant amplicons (major and minor allele) were extracted from an ethidium bromide-stained agarose gel using a QIAquick Spin kit (QIAGEN) and verified by sequencing (Mayo Clinic, Rochester) to ensure no other variants were present in the clone. Since v685 and v687 are only 8 bp apart, it was unavoidable that both variants would be present in the same amplicon. As a result, sequencing was used to ensure that both v685 major and minor allele test amplicons contained the major allele at v687 and both v687 amplicons contained the major allele at v685. The attB-flanked fragments were integrated via bacterial recombination into a kanamycin-resistant pDONR 221 vector using the BP Clonase II system (Invitrogen) to produce an entry clone. Entry clones were transformed into Library efficiency DH5α chemically competent E.coli (Invitrogen) and grown on Luria broth (LB) agar containing 50 µg/ml kanamycin overnight at 37°C. Single colonies were picked for inoculation in liquid LB broth containing 50 µg/ml kanamycin and incubated overnight in a shaking incubator at 37°C. Plasmids were extracted from the bacterial cells using a QIAprep spin kit (QIAGEN). Final expression clones were constructed by recombination of the entry clones with ampicillin resistant pGL3 promoter vector using the LR Clonase II system (Invitrogen). Expression clones were transformed into DH5α E.Coli and grown on LB agar containing 100 µg/ml ampicillin and single colonies were inoculated in LB broth containing 100 µg/ml ampicillin. Plasmids were extracted using endotoxin-free Zyppy Plasmid miniprep kit (Zymo research) and verified by sequencing. Four expression clones per variant were made in total; (1) major and (2) minor allele sequence cloned 5″ to the SV40 promoter and *Firefly Luciferase*, and (3) major and (4) minor allele sequence cloned 3″ to *Firefly Luciferase* gene.

**Table 2:** *IDE* variant specific primer sequences.

vID	rs number	Sense primer (5′ to 3′)	Antisense primer (5′ to 3′)	Size	Major	Minor	AT (°C)
685	rs11187061	GCAAGCAACAAGGATTGGAT	CCAAATTGCATGTCTGTAGCA	371	C	T	64
10	rs4646958	GCCTCAAGCCAAAACAAAAA	TCACTTCAGGCTGCAAAGAA	143	A	T	62
315	rs7895832	GCTGGAGAGTTATAAAAATAGCATTG	TTGTGGAAAGAAATTTACTATTTTGG	118	T	A	63
687	rs17107734	GCAAGCAACAAGGATTGGAT	CCAAATTGCATGTCTGTAGCA	371	C	T	64
154	rs4646957	GAGGCAGCCATGAAAGTTAGG	GGTGGTTGAGGTTTTAGCAATC	204	C	T	61

Primers specific for sequences in *IDE* surrounding each variant were used to generate attB- tagged products for cloning; sequences were amplified from DNA taken from individuals known to be homozygous for the major and minor allele indicated in the “Major” and “Minor” columns; vID; variant identification number, rs number; dbSNP variant identifier, Size; PCR product size (base pairs), AT; annealing temperature used for the amplification of each sequence.

### Cell Culture

Human neuroblastoma (Be(2)-C) and hepatocellular carcinoma (HepG2) immortalized cell lines were supplied by ATCC. Cells were cultured in Eagle Minimum Essential Medium (EMEM) supplemented with 10% fetal bovine serum, 2 mM L-Glutamine, 1X non-essential amino acids, 1000 U/ml Penicillin-Streptomycin (Sigma), 2.5 µg/ml Fungizone (Invitrogen). All cultures were incubated at 37°C in 5% CO2.

### Transfection

1×105 Be(2)-C cells or 3×105 HepG2 cells were plated in 12-well culture plates 24 hours before transfection. Cells were co-transfected in triplicate with the *IDE* expression clones and a pRL vector (Promega) containing the *Renilla Luciferase* reporter gene. Control wells included cotransfection of either pGL3 B (empty pGL3vector) or P (containing the SV40 promoter) with pRL. On day of transfection, cells were washed twice with PBS and media was replaced with 400 µl serum-free EMEM containing 200 ng expression clone or control vector, 10 ng pRL and transfection reagent Tfx-20 (Promega) at a charge ratio of 3∶1 (Tfx:DNA) per well. Transfection mix was pre-incubated for 15 minutes at room temperature. One hour after transfection, 800 µl complete EMEM was added to each well.

### Dual Luciferase Reporter Assay

Cells were washed twice with PBS and harvested with 200 µl of 1x Lysis buffer (Promega) for 20 minutes on a rocking platform 48 hours after transfection. The resultant lysates (5 µl) were plated in a white 96-well assay plate. Firefly Luciferase and Renilla Luciferase signals were measured by a Veritas microplate luminometer (Turner Biosystems) using the Dual Luciferase Reporter Assay System (Promega).

### Statistical Analyses

The ratio of Firefly Luciferase to Renilla Luciferase activity (Relative Luciferase Activity; RLA) was used to normalize Luciferase activity for intra-experimental transfection efficiency. Unpaired t-tests were used to compare mean RLA for all expression clones. Relative RLA (RRLA) to pGL3P was expressed as RLA for each *IDE* sequence as a percentage of RLA for pGL3P. The mean RRLA values for the major or minor allele that comprise each haplotype were used to generate a “Haplotype RRLA”. “Haplotype RRLA” for each haplotype were compared to H1 using paired t-tests. All statistics were performed using StatsDirect 2.6.1. Promoter sequences for *IDE* were identified using the ElDorado function of the Genomatix database (www.genomatix.de). Putative transcription factor binding sites were searched for *in silico* using the MatInspector tool of the Genomatix database; the DNA sequence of the regions surrounding the major and minor allele of each variant were compared for the presence of binding sites of known human transcription factors.

## Results

Each *IDE* sequence (surrounding either the major or minor allele of each variant) was cloned in two locations of the pGL3P vector; 5′ to the SV40 promoter and 3′ to *Firefly Luciferase* in order to see if the putative function of the variant would be specific to its position relative to the gene. As shown in Table 1, all the variants tested here are either intronic or lie in the 3′ flanking region. Since none of these variants lie within 1.5 kb of a promoter region (as defined by the ElDorado function at genomatix, www.genomatix.de), all variants were tested for enhancer rather than promoter activity. Classically, you might expect intronic enhancer or flanking sequences to work in both orientations (5′ or 3′ to the reporter gene) and that a genuine functional effect would be seen independent of where it was cloned. We transfected these vectors into a neuroblastoma cell line (Be(2)-C), and in order to assess tissue specificity, we also transfected the same vectors into a hepatocellular carcinoma cell line (HepG2).

**Figure 1:**
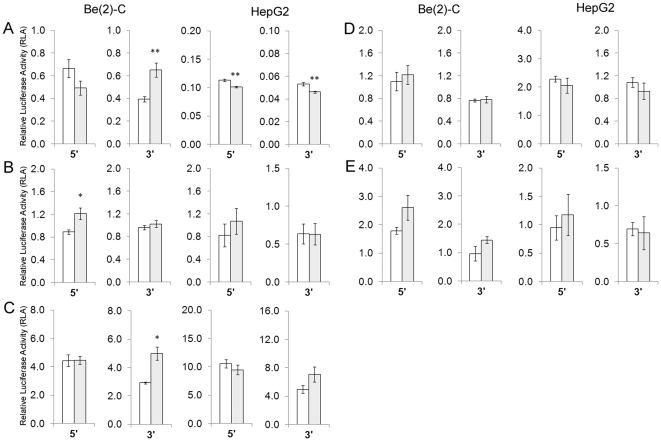
Functional effects of sequences surrounding five *IDE* variants on *in vitro* reporter gene expression. Relative Luciferase Activity (RLA; relative *Firefly:Renilla Luciferase* values) is shown for A) v685, B) v10, C) v315, D) v687 and E) v154 in Be(2)-C and HepG2 cell lines. Constructs containing sequence surrounding major allele are shown as white bars and those containing sequence surrounding minor allele are shown as shaded bars. *IDE* sequences were cloned either 5′ to the promoter or 3′ to *Firefly Luciferase*. Error bars represent standard error of the mean. * p = 0.05−0.01, ** p = 0.009−0.0001.

As shown in Figure 1a, contrasting association was observed for v685 major allele sequence compared to minor allele sequence in Be(2)-C and HepG2 cells. In Be(2)-C cells, although v685 minor allele sequence showed a non significant 0.82-fold RLA when cloned 5′ to the promoter (p = 0.82), a significantly 1.65-fold RLA was observed when cloned 3′ to the reporter gene (p = 0.009). In the HepG2 cells, a significant decrease in RLA was associated with the minor allele in both locations (5′ = 0.89-fold, p = 0.003; 3′ = 0.87-fold, p = 0.007), thus providing consistent association independent of cloning location and demonstrating a better correlation with decreased cerebellar *IDE* mRNA levels than Be(2)-C cells. An *in silico* search for putative transcription factor binding sites created or destroyed by the v685 minor allele (T) revealed the destruction of a site for PAX8. The function of this factor in the liver or brain is unknown, therefore we are unable to assess whether this may exert the tissue-specific effects we observed for this variant.

Figure 1b shows that v10 minor allele sequence was associated with 1.36-fold RLA compared to v10 major allele sequence albeit only when positioned 5′ to the promoter and only in Be(2)-C neuroblastoma cells (p = 0.02). Although a similar 1.31-fold RLA was observed for v10 in HepG2 hepatocellular cells at the 5′ location, the association was not significant (p = 0.22). Notably, we found that the v10 minor allele (T) destroys a putative binding site for MYT1, a repressor with brain and central nervous system specificity, which could explain the tissue-specific enhanced expression that we observed for this variant.

As shown in Figure 1c, in Be(2)-C cells, v315 minor allele sequence was also significantly associated with 1.71-fold RLA when compared to v315 major allele sequence albeit when positioned 3′ to the *Firefly Luciferase* reporter gene only (p = 0.02). A non-significant 1.42-fold RLA was observed in HepG2 cells in the 3′ location (p = 0.16). A search for putative transcription factor binding site at this locus revealed the destruction of an HMBOX1 site and the creation of TBP and VDR sites associated with the minor allele (A). HMBOX1 and VDR both function in the liver but have no known effects in the brain. The tissue-specificity of TBP is unknown. Since, significant association of v10 or v315 with *in vitro* reporter gene expression was inconsistent between cloning locations or between cell lines, we provide little evidence that v10 or v315 individually drive altered gene expression using this *in vitro* system. Figures 1d-e show that neither v687 nor v154 minor allele sequence were significantly associated with altered RLA in either location in either cell line when compared to major allele sequence (all p>0.07).

**Figure 2:**
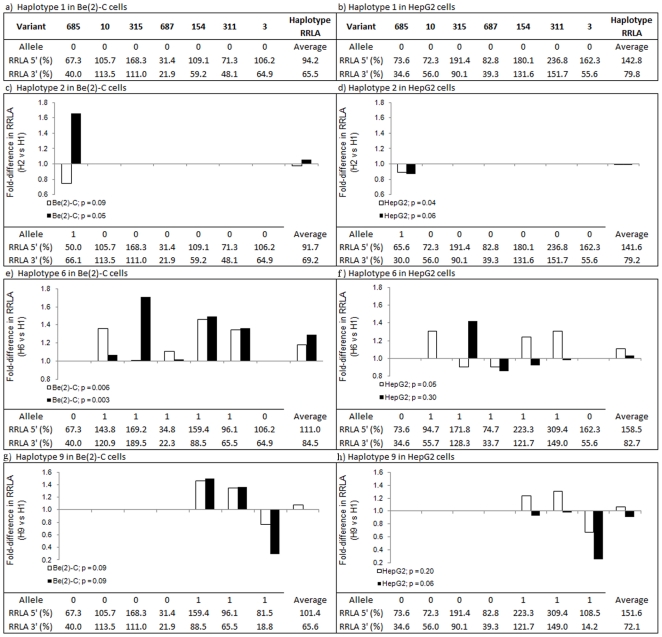
Relative RLA of *IDE* putative functional variants and haplotypes. Relative RLA levels to pGL3P (RRLA), expressed as % pGL3P expression are tabulated for each variant comprising a–b) H1, c–d) H2, e–f) H6, g–h) H9. 0 = major allele, 1 = minor allele. H1 = the most common *IDE* haplotype comprising the major allele at each variant. The RRLA for each sequence comprising haplotypes H2, H6 and H9 are plotted relative to the sequence comprising H1, expressed as fold-change in RRLA relative to H1. “Haplotype RRLA” is the averaged % RRLA for the variants in each haplotype. All standard error of the mean RRLA <0.7. P-values resulting from paired t-tests comparing “Haplotype RRLA” values for H2, H6 or H9 vs H1 are also shown.

These data provide an indication of how *IDE* variants exert effects on gene expression *in vitro* at the individual variant level. However, in addition to the individual variant effects, our previous publication reported altered *IDE* mRNA expression in post-mortem cerebellum between haplotypes. These haplotype-specific expression levels may be caused by an individual haplotype-tagging variant that is capable of altering gene expression levels to an extent that it can either drive the overall expression levels or can overcome any opposing effects of other variants present on the haplotype. Alternatively, a combination of many functional variants may act collectively to result in an overall increase or decrease in gene expression and will therefore only be detected by collecting data from multiple variants on the same haplotype. In an attempt to better understand how these variants may affect gene expression at the haplotypic level we first calculated the relative RLA (RRLA) to pGL3P. Since pGL3P contains the SV40 promoter but no *IDE* sequence, the RRLA provides a measure of whether the *IDE* sequence exerts enhanced or repressed RLA. RRLA was calculated for each major and minor allele sequence for the five variants tested here as well as for v311 and v3 included in our previous publication. The combined RRLA values for variants comprising H2 (minor allele at v685), H6 (minor allele at v10, v315, v687, v154 and v311) and H9 (minor allele at v154, v311 and v3) were then compared to the most common haplotype, H1 (major allele of each variant). The RRLA values and the composition of each haplotype (0 = major allele, 1 = minor allele) are tabulated in Figure 2. The RRLA values for sequences comprising H2, H6 and H9 were then plotted as fold-change in RRLA for the equivalent sequence in H1 (Figure 2).

As shown in Figure 2c, when cloned 3′to *Firefly Luciferase* and transfected into Be(2)-C cells the v685 minor allele sequence comprising H2 (66.1%) was associated with 1.65-fold RRLA relative to the v685 major allele sequence that comprises H1 (40.0%). v685 is the only one of the seven variants we tested that differs between H2 and H1, despite this, the overall RRLA for sequence comprising H2 was associated with 1.06-fold RRLA relative to H1 (69.2% vs 65.5%; p = 0.05). In contrast, in HepG2 cells (Figure 2d) the v685 minor allele was associated with near identical decreases in RRLA for the 5′ and 3′ constructs (0.89 and 0.87-fold, respectively) compared to the major allele sequences comprising H1. The altered expression at v685 alone was sufficient to provide a significant association with 0.99-fold RRLA for H2 sequences compared to H1 sequences at the 5′ location (141.6% vs 142.8%; p = 0.04). The data from HepG2 cells therefore correlated better with the decreased cerebellar *IDE* transcript levels than Be(2)-C cells.

In Be(2)-C cells, the sequences comprising the H6 haplotype were all associated with either equal or enhanced RRLA when compared to the sequences comprising H1 (Figure 2e). When cloned 5′ to the SV40 promoter, the minor allele sequences associated with the greatest fold change in RRLA relative to major allele sequences comprising H1 were v10 (1.36-fold), v154 (1.46-fold) and v311 (1.35-fold), whereas when cloned 3′ to the SV40 promoter, it was v315 (1.71-fold), v154 (1.50-fold) and v311 (1.36-fold). Overall, the altered expression associated with the sequences comprising H6 at both 5′ and 3′ location was significantly increased (1.18-fold and 1.29-fold, respectively) compared to the sequences comprising H1 (111.0% vs 94.2%; p = 0.006 and 84.5% vs 65.5%; p = 0.003, respectively). In HepG2 cells, a similar association was observed (Figure 2f); v10 (1.31-fold), v154 (1.24-fold) and v311 (1.31-fold) minor allele sequences drove an overall significant 1.11-fold RRLA associated with H6 compared to sequences comprising H1 although only at the 5″ location (158.5% vs 142.8%; p = 0.05). These data provide support for the hypothesis that multiple variants (v10, v154, v315 and v311) may contribute to the increased *IDE* transcript levels observed from post-mortem cerebellum samples.

In accordance with the fact that v154 and v311 comprise both H6 and H9, the same result was observed for these two variants in H9 (Figure 2g-h). In addition to v154 and v311, H9 comprises the v3 minor allele sequence associated with 0.77-fold and 0.29-fold RRLA when cloned in the 5′ and 3′ locations respectively in Be(2)-C cells and 0.67-fold and 0.26-fold RRLA in HepG2 cells. Although v154 and v311 consistently showed an increase in RRLA, the effect observed with the minor allele of v3 led to an overall RRLA associated with sequence comprising H9 that was not significantly altered compared to RRLA associated with sequence comprising H1. These data suggest that v154 and v311 may be the variants driving the increased gene expression we observed for H6 in post-mortem cerebellum samples.

## Discussion

Here we have tested five *IDE* variants for altered reporter gene expression and evaluated their contribution to the altered cerebellar *IDE* mRNA associations reported for the haplotypes they comprise. We have attempted to address the complex issue of how individual variants within a haplotypic backbone contribute to the “global” functional effect of that haplotype rather than solely concentrating on each variant as a singleton.

Firstly, the minor allele at v685, was significantly associated with 0.89-fold and 0.87-fold RLA when cloned 5′ to the promoter and 3′ to *Firefly Luciferase* in HepG2 cells, respectively.

However, these associations were not replicated in Be(2)-C cells, more than likely indicating tissue-specific differences in functionality. Likewise, when we compared the RRLA for the sequences comprising H2 for all seven *IDE* variants for which we have dual Luciferase Assay data with the RRLA for the sequences comprising the most common haplotype (H1), we found the same conflicting results between Be(2)-C and HepG2. Based on the assumption that Be(2)-C neuroblastoma cells correlate better with the cells taken from the cerebellum samples than the HepG2 hepatocytoma cells, these findings provide little evidence that v685 is the functional variant contributing to the significant decrease in *IDE* mRNA levels.

Five of the variants studied (v10, v315, v687, v154, v311) are present on the sixth most common *IDE* haplotype, H6. Due to the lack of consistency in association between cell lines and cloning locations for all except v311, we have little evidence that these variants can individually drive altered gene expression. Instead we show that when comparing the RRLA for the sequences comprising H6 vs H1, variants v10, v315, v154 and v311 (but not v687) contributed towards increased RLA associated with H6.

Along with v3, the variants, v154 and v311 occur on H9, a haplotype significantly (p = 0.006) associated with 2.34-fold *IDE* mRNA in post-mortem cerebellum samples. We found no significant difference in RRLA between H9 sequences and H1 sequences. This suggests that if there is a true association of H9 with *IDE* mRNA levels, it may be contributed to by variants other than v311 and v154, which were not sufficient to significantly increase the RRLA.

There are some provisos to the reporting of a lack of functionality associated with variants using the *in vitro* system incorporated here. (1) Due to LD within the region, the variants tested may tag the truly functional *IDE* variant; e.g. v776 is in perfect LD with v685 (D'and r^2^ = 1.0). In addition, one other variant in H2 (rs11187059) was not investigated in our association studies due to strong LD with v685 (r2 = 0.97). We are unaware of any other variant lying in a conserved region of *IDE* that is present on this haplotype. Regarding H6 and H9, only one variant in conserved regions remains untested (v180) due to its presence on multiple haplotypes. (2) Haplotypes may appear functional in opposing directions to another haplotype (eg. H2 vs H6) merely because they possess the opposing allele of a truly functional variant. (3) The *in vitro* model may not accurately reflect the complexity of *in vivo IDE* regulation. A larger sample size may be required to gain substantial power to detect mild effects on gene expression.

Experimental error may also confound attempts to observe these effects; we calculated the coefficient of variation (%CV; standard deviation RLA/mean RLA*100) as a statistic for comparing the degree of variation in RLA across triplicates. In the 210 transfections performed, the %CV was <25% in 93.3% of the transfections and always <40%. Although a %CV <25% indicates relatively little variation for an *in vitro* study, it may be sufficient to reduce the power to detect subtle effects on RLA. Furthermore, it is well recognized that cis-regulatory function is context dependent [16]. These experiments were conducted under basal conditions and would therefore not detect altered gene expression as a result of stimulation by inflammation or oxidative stress that may occur in the AD brain. In addition, a regulatory variant may require interaction with intronic sequence or other downstream regulatory elements to exert its function [16], thus highlighting a limitation of the Dual Luciferase Assay, which allows only small areas of the genome to be tested at once. (4) Variants may exert effects via a mechanism other than altered transcription factor binding, e.g. by affecting mRNA processing, pre-mRNA splicing, mRNA stability, mRNA trafficking, or regulatory RNAs. (5) The association of variants with altered *IDE* mRNA previously observed may have been a false positive finding; it must be noted that the p-value for association of v685 with altered *IDE* transcript (p = 0.009) just missed the Bonferroni corrected significance level (p = 0.005) for the ten variants tested. We currently do not have sufficient evidence to distinguish between these possibilities.

In summary, we have identified multiple, functional variants residing in conserved, non-coding *IDE* sequence that alter reporter gene expression *in vitro*, in a direction consistent with association with *IDE* mRNA transcript in post-mortem cerebellum samples. Since IDE has been shown to mediate Aβ degradation *in vitro* [5]-[7], variants that regulate *IDE* expression could represent good therapeutic targets for LOAD.

## References

[1] Roher AE, Kasunic TC, Woods AS, Cotter RJ, Ball MJ (1994). Proteolysis of A beta peptide from Alzheimer disease brain by gelatinase A.. Biochem Biophys Res Commun.

[2] Backstrom JR, Lim GP, Cullen MJ, Tokes ZA (1996). Matrix metalloproteinase-9 (MMP-9) is synthesized in neurons of the human hippocampus and is capable of degrading the amyloid-beta peptide (1-40).. J Neurosci.

[3] Iwata N, Tsubuki S, Takaki Y, Watanabe K, Sekiguchi M (2000). Identification of the major Abeta1-42-degrading catabolic pathway in brain parenchyma: suppression leads to biochemical and pathological deposition.. Nat Med.

[4] Hu J, Igarashi A, Kamata M, Nakagawa H (2001). Angiotensin-converting enzyme degrades Alzheimer amyloid beta-peptide (A beta ); retards A beta aggregation, deposition, fibril formation; and inhibits cytotoxicity.. J Biol Chem.

[5] Kurochkin IV, Goto S (1994). Alzheimer's beta-amyloid peptide specifically interacts with and is degraded by insulin degrading enzyme..

[6] McDermott JR, Gibson AM (1997). Degradation of Alzheimer's beta-amyloid protein by human and rat brain peptidases: involvement of insulin-degrading enzyme..

[7] Qiu WQ, Walsh DM, Ye Z, Vekrellis K, Zhang J (1998). Insulin-degrading enzyme regulates extracellular levels of amyloid beta-protein by degradation..

[8] Farris W, Mansourian S, Chang Y, Lindsley L, Eckman EA (2003). Insulin-degrading enzyme regulates the levels of insulin, amyloid beta -protein, and the beta -amyloid precursor protein intracellular domain in vivo..

[9] Bernstein HG, Ansorge S, Riederer P, Reiser M, Frolich L (1999). Insulindegrading enzyme in the Alzheimer's disease brain: prominent localization in neurons and senile plaques.. Neurosci Lett.

[10] Blacker D, Bertram L, Saunders AJ, Moscarillo TJ, Albert MS (2003). Results of a high-resolution genome screen of 437 Alzheimer's disease families.. Hum Mol Genet.

[11] Kehoe P, Wavrant-De Vrieze F, Crook R, Wu WS, Holmans P (1999). A full genome scan for late onset Alzheimer's disease.. Hum Mol Genet.

[12] Bertram L, Blacker D, Mullin K, Keeney D, Jones J (2000). Evidence for genetic linkage of Alzheimer's disease to chromosome 10q.. Science.

[13] Prince JA, Feuk L, Gu HF, Johansson B, GatzM (2003). Genetic variation in a haplotype block spanning IDE influences Alzheimer disease.. Hum Mutat.

[14] Ertekin-Taner N, Allen M, Fadale D, Scanlin L, Younkin L (2004). Genetic variants in a haplotype block spanning IDE are significantly associated with plasma Abeta42 levels and risk for Alzheimer disease.. Hum Mutat.

[15] Carrasquillo MM, Belbin O, Zou F, Allen M, Ertekin-Taner N (2010). Concordant association of insulin degrading enzyme gene (IDE) variants with IDE mRNA, Abeta, and Alzheimer's disease..

[16] Rockman MV, Wray GA (2002). Abundant raw material for cis-regulatory evolution in humans..

